# Clinical value of total white blood cells and neutrophil counts in patients with suspected appendicitis: retrospective study

**DOI:** 10.1186/1749-7922-7-32

**Published:** 2012-10-02

**Authors:** Zuhoor K Al-gaithy

**Affiliations:** 1 Department of Surgery, King Abdulaziz University, P.O. Box 80215, Jeddah, 21589, Saudi Arabia

**Keywords:** Acute appendicitis, Diagnosis, White blood cells, Histological diagnosis, Neutrophil count, Receiver operating characteristic curves

## Abstract

**Introduction:**

Acute appendicitis (AA) is common surgical problem associated with acute-phase reaction. Blood tests role in decision-making process is unclear. This retrospective study aimed to determine diagnostic value of preoperative evaluation of white blood cells (WBCs) and neutrophils and its value in predicting AA severity.

**Methods:**

Medical records of 456 patients who underwent appendectomy during 4-years period were retrospectively reviewed. Patients were subdivided according to histological finding into: normal appendix (n = 29), uncomplicated inflamed appendix (n = 350), complicated appendicitis (n = 77). Diagnostic performances of WBCs and neutrophils were analyzed using receiver operating characteristic (ROC) curves.

**Results:**

WBCs and neutrophils counts were higher in patients with inflamed and complicated appendix than normal appendix and in complicated than inflamed appendix. In patients, WBCs count 9.400 × 10^3^/mL had sensitivity of 76.81%, specificity of 65.52%, positive predictive value (PPV) of 97.0%, negative predictive value (NPV) of 16.1%, positive likelihood ratio [LR(+)] of 2.23, negative LR(−) of 0.35. Neutrophil count 7.540 × 10^3^/mL had sensitivity of 70.96%, specificity of 65.52%, PPV of 96.8%, NPV of 13.3%, LR(+) of 2.06, LR(−) of 0.44. Areas under ROC curve were 0.701, 0.680 for elevated WBCs and neutrophils count.

**Conclusions:**

Clinicians should not rely on either elevated WBCs or neutrophils count as appendicitis indicator as clinical data are superior in decision-making appendectomy.

## Introduction

Acute appendicitis (AA) is one of the most common abdominal emergencies. Although patients with AA often present with a characteristic symptom complex and physical findings, atypical presentations are common. Missed or delayed diagnosis can lead to increased rates of perforation and morbidity [1]. The clinical diagnosis of AA is difficult, and management errors are frequent, with rates of negative explorations reaching 20% to 30% [2]. Despite the wide use of imaging techniques, appendicitis remains a challenging diagnosis [3].

Patients with suspected appendicitis are mainly managed on the basis of their disease history and physical examination; the value of laboratory examinations is controversial. Some works have assessed the diagnostic accuracy of different inflammatory markers in appendicitis with heterogeneous designs and results including: total white blood cells (WBCs), granulocytes, C-reactive protein, leukocyte elastase activity, D-lactate, phospholipase A2 and interleukine-6 [4-6]. Studies have shown inconsistent information regarding the use of WBCs count and differential in AA diagnosis. Although most studies show an association between elevated WBCs count in appendicitis diagnosis, its significance varies greatly [7-10]. Another question that has been raised is whether a normal WBCs count and differential can adequately rule out a diagnosis of appendicitis. There have been reports of high negative predictive values (NPVs >90%) for normal WBCs count and differential [7,9].

The aim of this retrospective study was to assess diagnostic value of total WBCs and neutrophils counts in patients who underwent appendectomy due to suspicious of AA. Using receiver-operating characteristic (ROC) curves, sensitivity, specificity, NPV, positive predictive value (PPV), and likelihood ratios (LR) were calculated by correlating the preoperative WBCs and neutrophil counts with histological diagnosis. In addition, this study will attempt to determine cutoff point for WBCs and neutrophils counts with best sensitivity and specificity for determination of acute appendicitis.

## Material and methods

Four hundred and fifty six patients (273 male and 183 female) who underwent appendectomy with a clinical diagnosis of AA in Surgery Department at King Abdulaziz Medical Center, Jeddah, Saudi Arabia were recruited in this retrospective study between January 2003 and January 2007. The diagnosis of AA was established by history, clinical examination, and laboratory tests including WBCs and neutrophil counts. Demographic, symptoms, signs, surgical procedures, and histopathological results of appendix examination were recorded. Patients who underwent incidental appendectomy as part of another procedure, and patients on steroids or immunosuppressive medications excluded from the study. According to the results of histopathological examination of the removed appendix, patients were divided into 3 groups, group (1) normal appendix (no pathological diagnosis) (n = 29); group (2) with uncomplicated inflamed appendicitis (n = 350) and group (3) with complicated appendicitis (n = 77) (perforated and gangrenous). The ethical committee of King Abdelaziz University approved the study.

Laboratory tests were carried on admission to hospital before antibiotics administered. WBCs count and differential were measured by an automated hematology analyzer counter (SE-9000; Sysmex, Kobe, Japan). All the excised appendices were underwent histopathological examination.

### Data analysis

The statistical analysis was performed using MedCalc for Windows, version 5.0 (MedCalc Software, Mariakerke, Belgium) and Statistical Package for the Social Sciences for Windows, version 12.0 (SPSS Inc., Chicago, IL, USA). The data were expressed as mean +/− stander deviation [SD] (range) or number (%) as appropriate. Statistical analysis was done with one-way analysis of variance to compare data between groups. For comparison of 2 groups unpaired Student ”t test” and Chi square test were used for parametric and non-parametric parameters, respectively. For describing the diagnostic properties of WBCs and neutrophils counts, we used the area under ROC curve (AUC) and likelihood ratio (LR) [11]. AUC of 1.00 indicates perfect discriminating power while area of 0.50 indicates absence of discriminating power. LR (+) is the ratio of the frequency of a finding among the diseased patients (true-positive rate) and among the non-diseased patients (false-positive rate). A true diagnostic test usually has an LR >10, and an exclusion test has a LR < 0.1. All results were reported with 95% confidence intervals (95% CIs). A *P* value of < 0.05 was considered statistically significant.

## Results

Table 1 showed patients’ demographic characteristics. The number of males were significantly higher than females (273 versus 183, *P* < 0.0001). Regarding type of operation, 406 patients underwent opened appendectomy, 45 patients had laparoscopic appendectomy and 5 had laparoscopic converted to open with significant difference between them *P* < 0.0001.

**Table 1:** Demographic characteristics of the patients

**Parameters**	**All patients**
	**(*n*** **= 456)**
**Age** (years)	23.25 ± 9.80
	(6.00-61.00)
**Gender**	
Male	273 (59.9%)
Female	183 (40.1%)
Significance	***P*** **< 0.0001**
**Operation type**	
Open	406 (89.0%)
Laparoscopic	45 (9.9%)
Laparoscopic converted to open	5 (1.1%)
Significance	***P*** **< 0.0001**

Data are expressed as mean +/− SD (range) or number (%). Significant between variables was made using non parametric Chi-Square test.

Table 2 showed the clinical and laboratory characteristics of patients subgroups according to the hisopathological findings. In normal, inflamed and complicated appendix, the type of pain was mainly localized 88,2%, 82.7%, 68.8% than generalized 13.8%, 18.3%, 31.2% with significant difference between groups *P* < 0.026. In normal, inflamed and complicated appendix, the duration of pain was mainly >12 hours, 75.9%, 88.3%, 98.7% than ≤12 hours, 24.1%, 11.8%, 1.3% with significant difference between patients subgroups *P* < 0.002. Fever was significantly higher in complicated than normal or inflamed appendix (64.9% versus 24.1% and 47.7%, *P* < 0.0001). WBCs and neutrophils counts were higher in inflamed (*P* < 0.019, *P* < 0.045) and complicated (*P* < 0.001, *P* < 0.001) than normal appendix and in complicated than inflamed appendix (*P* < 0.045, *P* < 0.004).

**Table 2:** Clinical and laboratory characteristics of patient subgroups

**Parameters**	**Normal appendix**	**Appendicitis (*n*= 427, 93.6%)**		***P*-Value**
	**(*n*** **= 29, 6.4%)**			
		**Inflamed**	**Complicated**	
		**(n = 350, 76.8%)**	**(n = 77, 16.9%)**	
**Pain type**				**0.026**
Localized	25 (88.2%)	286 (81.7%)	53 (68.8%)	
Generalized	4 (13.8%)	64 (18.3%)	24 (31.2%)	
**Pain duration**				**0.002**
≤12 hours	7 (24.1%)	41 (11.8%)	1 (1.3%)	
>12hours	22 (75.9%)	309 (88.3%)	76 (98.7%)	
**Symptoms & signs**				
Vomiting	18 (62.1%)	268 (76.6%)	64 (83.1%)	0.072
Anorexia	17 (58.6%)	261 (74.6%)	54 (70.1%)	0.151
Nausea	14 (48.3%)	193 (55.1%)	44 (57.1%)	0.713
Fever	7 (24.1%)	167 (47.7%)	50 (64.9%)	**0.0001**
Diarrhea	2 (6.9%)	17 (4.9%)	3(3.9%)	0.812
Dysurea	2 (6.9%)	8 (2.3%)	4 (5.2%)	0.190
**Laboratory investigations**				
**WBCs count** (× 10^3^/mm^3^)	10.67 ± 7.56	13.03 ± 4.94	14.34 ± 5.25	
	(4.10-35.70)	(2.90-29.60)	(2.20-33.60)	
*Significance		*P<0.019	*P<0.001,**P<0.045	
**Neutrophil count** (× 10^3^/mm^3^)	7.95 ± 6.67	9.92 ± 4.88	11.74 ± 4.88	
	(1.10-30.93)	(0.20-27.10)	(1.70-24.67)	
*Significance		*P<0.045	*P<0.001,**P<0.004	

Data are expressed as mean +/− SD (range) or number (%). Significant between subgroups was made using Chi-Square test (P) for non-parametric parameters and *ANOVA test for parametric parameters, *P* significance between all groups, *P significance versus controls, **P significance versus inflamed appendix.

**Figure 1:**
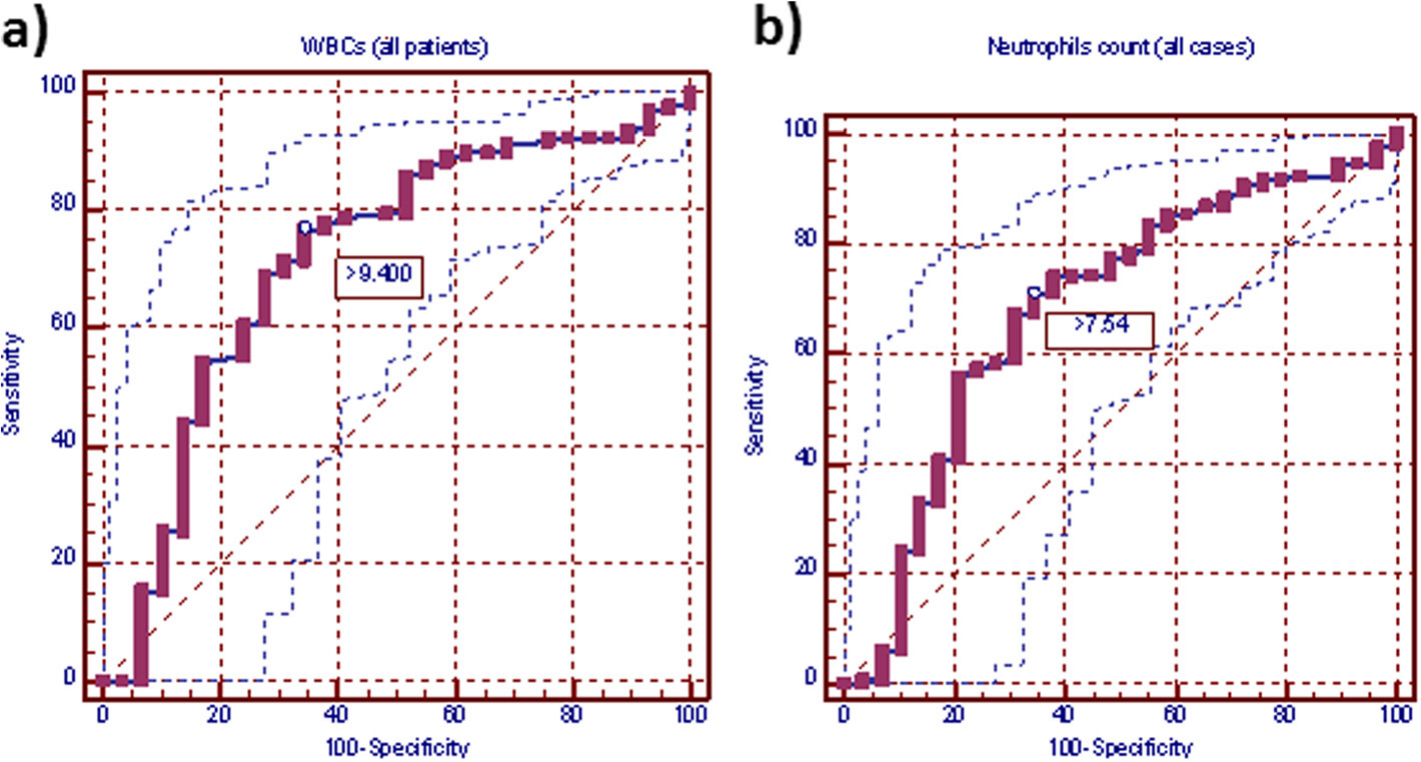
**Receiver-operating characteristic curve (ROC) for white blood cells and neutrophil counts in all appendectomy patients. a)** ROC for white blood cells in all appendectomy patients. ROC for white blood cell count of all appendectomy patients. Area under the curve (AUC) was 0.701 (standard error, 0.055; 95% CI =0.671-0.755). Ideal white blood cell count cutoff value was 9,400 cells/mm^3^, this yields sensitivity of 76.8% and specificity of 65.5%. **b)** ROC for neutrophils count of all appendectomy patients. AUC is 0.680 (standard error, 0.056; 95% CI = 0.635-0.722). Neutrophils count ideal cutoff value was 7.540 ×10^3^ cells/mm^3^, this yields sensitivity of 71.0% and specificity of 65.5%.

**Figure 2:**
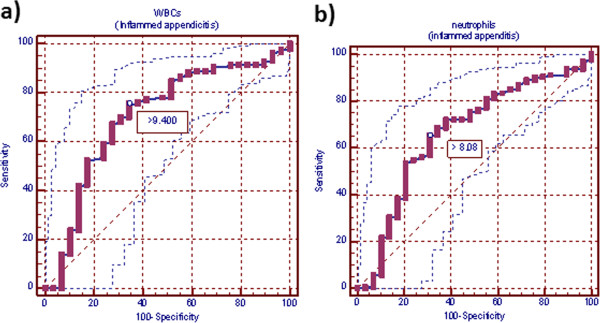
**Receiver-operating characteristic curve (ROC) for white blood cells and neutrophil counts in inflamed appendicitis patients. a)** ROC for white blood cells in inflamed appendicitis patients. Area under curve (AUC) is 0.704 (standard error, 0.055; 95% CI =0.655-0.749). White blood cell count ideal cutoff value was 9,400 ×10^3^ cells/mm^3^; this yields sensitivity of 75.4% and specificity of 65.5%. **b)** ROC for neutrophils count in inflamed appendicitis patients. AUC was 0.664 (standard error, 0.056; 95% CI = 0.614-0.712). Neutrophils count ideal cutoff value was 8.080 × 10^3^ cells/mm^3^, this cutoff value yields sensitivity of 65.4% and specificity of 69.0%.

**Figure 3:**
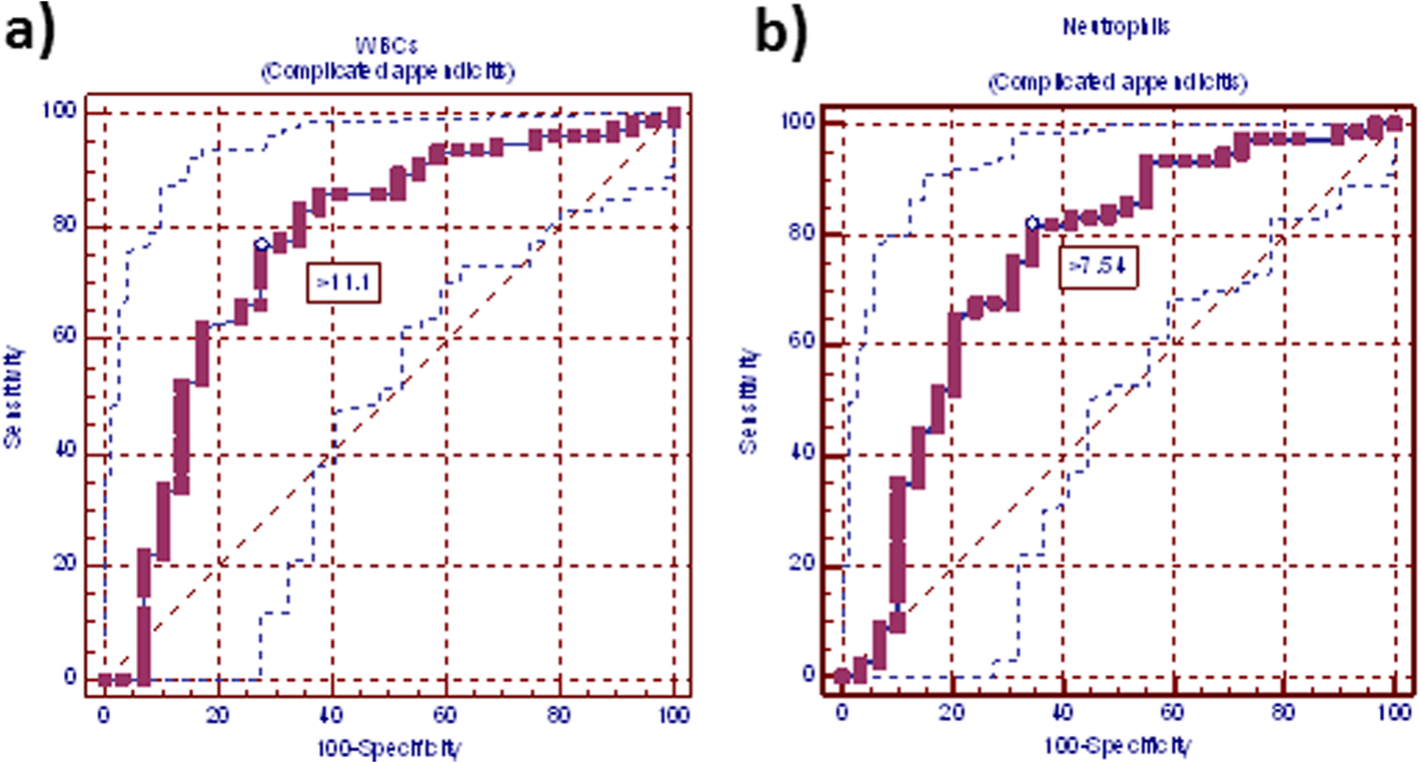
**Receiver-operating characteristic curve (ROC) for white blood cells and neutrophil counts in complicated appendicitis patients. a)** ROC curve for white blood cell count in complicated appendicitis patients. Area under curve (AUC) was 0.763 (standard error, 0.058; 95% CI = 0.670-0.840). White blood cell count ideal cutoff value was 11.100 × 10^3^ cells/mm^3^, this cutoff value yields sensitivity of 75.4% and specificity of 65.5%. **b)** ROC curve for neutrophils count in complicated appendicitis patients. AUC was 0.749 (standard error, 0.060; 95% CI = 0.656-0.828). Neutrophils count ideal cutoff value was 7.540 × 10^3^ cells/mm^3^, this cutoff value yields sensitivity of 81.8% and specificity of 65.5%.

Cut-off values, at which the greatest sum of sensitivity and specificity was obtained, in WBCs and neutrophils counts were 9.400×10^3^ and 7.540×10^3^, respectively in all patients with appendicitis versus normal appendix; 9.400×10^3^ and 8.080 ×10^3^, respectively in patients with inflamed versus normal appendix and 11.100×10^3^ and 7.540×10^3^, respectively in patients with complicated versus normal appendix. At these cutoff points, sensitivity, specificity, PPV, NPV, LR (+) and LR (−) for WBCs and neutrophils were for normal versus all abnormal appendix for WBCs: 76.81, 65.52%, 97.0%, 16.1%, 2.23%, 0.35%; for neutrophils: 70.96%, 65.52%, 96.8%, 13.3%. 2.06%. 0.44%; for normal versus inflamed appendix for WBCs: 75.43%, 65.52%, 96.4%, 18.1%, 2.19%, 0.38%; for neutrophils: 65.43%, 68.97%, 96.2%. 14.2%, 2.11, 0.50%; for normal versus complicated appendix for WBCs: 76.62%, 72.41%, 88.10%, 53.80%, 2.78%, 0.32%; for neutrophils: 81.82%, 65.52%, 86.30%. 57.60%, 2.37, 0.28% (Table 3; Figures 1, 2 and 3).

**Table 3:** Performance characteristics estimate of normal versus different groups

**Parameters**	**Cutoff point**	**Sensitivity**	**Specificity**	**PPV**	**NPV**	**LR(+)**	**LR(−)**
**normal versus all abnormal appendix (*n*= 456)**							
**WBCs count** 95% CIs	9.400 X10^3^	76.81 (72.5 - 80.7)	65.52 (45.7 - 82.1)	97.0 (4.6 - 98.6)	16.1 (10.0 - 24.0)	2.23 (1.7- 2.9)	0.35 (0.2 - 0.6)
**Neutrophil** count 95% Cls	7.540X10^3^	70.96 (66.4 - 75.2)	65.52 (45.7 - 82.1)	96.8 (94.2 - 98.5)	13.3 (8.2 - 20.0)	2.06 (1.6 - 2.7)	0.44 (0.3 - 0.7)
**normal versus inflamed appendix (*n*= 379)**							
**WBCs count** 95% CIs	9.400 X10^3^	75.43 (70.6 - 79.8)	65.52 (45.7 - 82.1)	96.4 (93.4 - 98.2)	18.1 (11.2 - 26.9)	2.19 (1.7 - 2.9)	0.38 (0.2 - 0.6)
**Neutrophil** count 95% Cls	8.080X10^3^	65.43 (60.2 - 70.4)	68.97 (49.2 - 84.7)	96.2 (92.9 - 98.3)	14.2 (8.9 - 21.1)	2.11 (1.6 - 2.7)	0.50 (0.3 - 0.9)
**normal versus complicated appendix (*n*= 106)**							
**WBCs count** 95% CIs	11.100 X10^3^	76.62 (65.6 - 85.5)	72.41 (52.8 - 87.3)	88.10 (77.8 - 94.7)	53.80 (37.2 - 69.9)	2.78 (2.1 - 3.6)	0.32 (0.2 - 0.7)
**Neutrophil count** 95% Cls	7.540X10^3^	81.82 (71.4 - 89.7)	65.52 (45.7 - 82.1)	86.30 (76.2 - 93.2)	57.60 (38.9 - 74.8)	2.37 (1.8 - 3.2)	0.28 (0.1 - 0.6)

*WBCs* white blood cells, *95% CIs* 95% confidence intervals, *NPV* negative predictive value, *PPV* positive predictive value, *LR* likelihood ratio.

## Discussion

Although the incidence of AA appears to have been waning slightly over the past few decades, it remains a frequent cause of acute abdominal pain and urgent operative intervention. The analysis of a patient with possible appendicitis can be divided into 3 parts: history, physical examination, and routine laboratory and radiological tests. The pain was reported in 456 (100%) of our cases which was mostly localized than generalized and mostly more than 12 hours. In this respect, Mughal and Soomro [12] have noted pain in 66.7% of patients while, Soomro [13] reported abdominal pain in 98.27% of appendicitis patients. Pain involves whole abdomen when there is perforation leading to peritonitis [14]. This was also true in this series as in complicated appendicitis; generalized pain was more than in normal or inflamed appendicitis. In our cases, second most common presenting symptom was vomiting 76.8% followed by anorexia72.9%, nausea 55.0%, fever 49.1%, diarrhea 4.8% then dysuea 3.1%. Salari and Binesh [15] reported anorexia in 84.48% of patients in pediatric age group while, Soomro [13] reported anorexia in 86.20% of patients. At operation, we found 29 (6.4%) patients with normal appendix, 350 (76.8%) with inflamed appendix, 77 (16.9%) with complicated appendix. Soomro [13] reported that at operation 31 (53.44%) patients with simple appendicitis and 26 (44.82%) patients with complicated appendicitis. In literature the rate of perforated and gangrenous appendicitis has been quoted as 16-57% [14,16].

Acute appendicitis remains a challenging diagnosis. Almost one-third of patients have atypical clinical features. The wide use of ultra sonography and computer tomography scan has not effectively decreased the rate of perforated appendicitis or number of negative appendectomies in large population studies [3] despite the hopeful results of some case series in tertiary care academic hospitals [1,17]. Some authors have assessed the diagnostic value of inflammatory markers with varied designs and results [7,18-20]. Variety of designs explains the lack of evidence in the two meta-analysis published to date about inflammatory markers diagnostic utility [9,21]. Although, over the last few decades, several inflammation markers have been proposed to increase diagnostic accuracy in AA including phospholipase A2, [4] amyloid A, [22] leukocyte elastase, [23] neutrophil count, [9] several interleukins and cytokines, [24] WBCs and neutrophil counts are certainly the most widely used.

In this study, WBCs and neutrophil counts were significantly higher in patients with inflamed and complicated than normal appendix and in complicated than inflamed appendix. Several reports suggest that an elevated leukocyte count is usually the earliest laboratory test to indicate appendiceal inflammation, and most of the patients with acute appendicitis present with leukocytosis [25] despite several studies that acknowledge the limitations of this test [26,27]. Sack et al. [28].found that WBCs count was clearly elevated in children with phlegmonous and perforated appendicitis. Mughal and Soomro [12] found total leucocytes and neutrophil counts elevated in all their patients. Soomro [13] reported elevation of total leucocytes and neutrophils counts in 53.33% of their patients. Meanwhile, Yokoyama et al. [29] reported that WBCs counts and neutrophil percentage are not useful for surgical indication.

Previous studies assessing the relationship between WBCs count and appendicitis have their findings reported in a variety of ways, including comparing mean values for total WBCs count in patients with and without appendicitis, and variously using *P*-values, sensitivity, specificity, PPV and NPV [23,30]. These studies can be difficult to interpret, because both PPV and NPV depend on disease prevalence. Moreover, sensitivity and specificity alone do not allow clinicians to directly apply diagnostic tests results to individual patients. Grönroos et al. [4] were the first to report that an increased leukocyte count was a very early marker of appendiceal inflammation in adult patients, according to ROC analysis. Contrary to descriptive and comparing statistical methods, analysis of ROC curves allows the estimation and verification of diagnostic suitability of diagnostic parameters. LR(+) is defined as the true-positive rate over the false-positive rate. It allows the clinician to assess the likelihood that a patient with a given test result (i.e., elevated WBCs count) has that disease. Additionally, LR is independent of disease prevalence. Generally, a clinically useful diagnostic test has an LR >10 or <0.1.

In this study, cut-off values, at which greatest sum of sensitivity and specificity was obtained, in WBCs and neutrophils counts were (9.400×10^3^ and 7.540×10^3^, respectively) in all patients with appendicitis versus normal appendix. At these cutoff points, AUC (95% CI) for WBCs and neutrophils were 0.701 (standard error, 0.055; 95% CI = 0.671-0.755) and 0.680 (standard error, 0.055; 95% CI = 0.635-0.722). WBCs and neutrophils sensitivity were 76.81%, 70.96%, specificity 65.52%, 65.52%, PPV 97.0%, 96.8%, NPV 16.1%, 13.3%, LR(+) 2.23, 2.06 and LR(−) 0.35, 0.44. Meanwhile, when we took only cases with inflamed appendicitis versus normal appendix, cut-off values in WBCs and neutrophils counts were 9.400 ×10^3^ and 8.080 ×10^3^, respectively. At these cutoff points, AUC (95% CI) for WBCs and neutrophils were 0.704 (standard error, 0.055; 95% CI = 0.655-0.749) and 0.664 (standard error, 0.056 95% CI = 0.614-0.712). WBCs and neutrophils sensitivity were 75.43%, 65.43%, specificity 65.52%, 68.97%, PPV 96.4%, 96.2%, NPV 18.1%, 14.2%, LR(+) 2.19, 2.11 and LR(−) 0.38, 0.50. While, when we took only cases with complicated appendicitis versus normal appendix, cut-off values in WBCs and neutrophils counts were 11.100 ×10^3^ and 7.540 ×10^3^, respectively. At these cutoff points, AUC (95% CI) for WBCs and neutrophils were 0.763 (standard error, 0.058; 95% CI = 0.670 - 0.840) and 0.749 (standard error, 0.060; 95% CI = 0.656 - 0.828). WBCs and neutrophils sensitivity were 76.62%, 81.82%, specificity 72.41%, 65.52%, PPV 88.10%, 86.30%, NPV 53.80%, 57.60%, LR(+) 2.78, 2.37 and LR(−) 0.32, 0.28. ROC curve analysis of our data suggests that there is no value of WBCs or neutrophils counts that is sensitive and specific enough to be clinically useful. An ideal test has an AUC of 1, while a perfectly random test has an AUC of 0.5. Generally, a “good” test has an AUC >0.8 and an “excellent” test has an AUC >0.9. In this respect, it had been reported that inflammatory markers such as WBCs is poorly reliable in confirming the presence of AA because of their low specificity in adults and children [2,7,31]. Sensitivity and specificity for WBCs count determined in this study is comparable with various national [32,33] and international [6,33-35] studies in which sensitivity ranges from 80.0–88.7%, while specificity ranges from 61.5-87.0%. So, leukocyte count by itself is not completely preventive against negative appendectomy, a finding consistent with our results.

Other investigators have constructed ROC curves for WBCs count and appendicitis with similar results. Körner et al. [36] found AUC of 0.69 (95% CI = 0.65-0.73), statistically no different from our results. Grönroos et al. [4] found a AUC of 0.730 (standard error = 0.041). Rodriguez- Sanjuan et al. [37] found an AUC of 0.67 (standard error = 0.08) for WBCs count and appendicitis in children. Paajanen et al. [18] found an AUC of 0.76. Andersson et al. [38] found an AUC of 0.80 (standard error = 0.02) for patients admitted to hospital for suspected appendicitis. An elevated total WBCs count might erroneously lead a surgeon to operate when other features of clinical scenario do not warrant or alternatively delay intervention as a result of a normal WBCs count. In support, of Guss and Richards [39] showed an association between delay in operative intervention and higher rate of perforated appendix in patients presenting to emergency with eventual diagnosis of appendicitis and normal WBCs count.

### Limitations

The main limitation of this study that it is retrospective so there is biases in inclusion criteria of the patients which included all patients who underwent appendectomy, another prospective study containing all patients with abdominal pain with suspension of appendicitis must be made.

## Conclusion

Leukocyte and neutrophils counts should not be used as diagnostic criteria for acute appendicitis because of its low sensitivity and specificity and must depend on clinical data as they are superior in decision-making appendectomy. WBCs and neutrophils counts do not indicate disease severity. WBCs and neutrophils counts in appendicitis evaluation does not enhance clinical decision making. The sensitivity of these tests is insufficient to achieve reliable rule-out.

## Abbreviations

AA: Acute appendicitis; AUC: Area under curve; LR: Likelihood ratios; NPV: Negative predictive value; PPV: Positive predictive value; ROC: Receiver operating characteristic curves; WBCs: White blood cells.

## Competing interests

The authors declare that they have no competing interests.

## Authors' contributions

ZA carried out the design the study, collection and analysis of data, drafting and approved the final manuscript for publication.
